# Implementing palliative care education into primary care practice: a qualitative case study of the CAPACITI pilot program

**DOI:** 10.1186/s12904-023-01265-7

**Published:** 2023-09-28

**Authors:** Midori Matthew, Daryl Bainbridge, Valerie Bishop, Christina Sinding, Samantha Winemaker, Frances Kilbertus, Katherine Kortes-Miller, Hsien Seow

**Affiliations:** 1 Department of Health Research Methods, Evidence and Impact, McMaster University, Hamilton, ON Canada; 2 Department of Oncology, McMaster University, Hamilton, ON Canada; 3 School of Social Work, McMaster University, Hamilton, ON Canada; 4 Division of Palliative Care, McMaster University, Hamilton, ON Canada; 5 Northern Ontario School of Medicine University, Thunder Bay, ON Canada; 6 Department of Social Work, Lakehead University, Thunder Bay, ON Canada

**Keywords:** Palliative care, Knowledge translation, Primary care, Interprofessional education

## Abstract

**Background:**

CAPACITI is a virtual education program that teaches primary care teams how to provide an early palliative approach to care. After piloting its implementation, we conducted an in-depth qualitative study with CAPACITI participants to assess the effectiveness of the components and to understand the challenges and enablers to virtual palliative care education.

**Methods:**

We applied a qualitative case study approach to assess and synthesize three sources of data collected from the teams that participated in CAPACITI: reflection survey data, open text survey data, and focus group transcriptions. We completed a thematic analysis of these responses to gain an understanding of participant experiences with the intervention and its application in practice.

**Results:**

The CAPACITI program was completed by 22 primary care teams consisting of 159 participants across Ontario, Canada. Qualitative data was obtained from all teams, including 15 teams that participated in focus groups and 21 teams that provided reflection survey data on CAPACITI content and how it translated into practice. Three major themes arose from cross-analysis of the data: changes in practice derived from involvement in CAPACITI, utility of specific elements of the program, and barriers and challenges to enacting CAPACITI in practice. Importantly, participants reported that the multifaceted approach of CAPACITI was helpful to them building their confidence and competence in applying a palliative approach to care.

**Conclusions:**

Primary care teams perceived the CAPACITI facilitated program as effective towards incorporating palliative care into their practices. CAPACITI warrants further study on a national scale using a randomized trial methodology. Future iterations of CAPACITI need to help mitigate barriers identified by respondents, including team fragmentation and system-based challenges to encourage interprofessional collaboration and knowledge translation.

**Supplementary Information:**

The online version contains supplementary material available at 10.1186/s12904-023-01265-7.

## Introduction

A palliative approach to care emphasises early involvement of palliative care beginning upon diagnosis of an incurable illness or progression of a serious illness, [1] incorporating the World Health Organization’s (WHO) definition of this care as a holistic approach that improves quality of life of patients and their families facing problems associated with serious illness through prevention and relief of suffering and treatment of distressing symptoms [2]. A palliative approach to care has been demonstrated to improve patient and family outcomes when used by community-based primary health care teams [3, 4]. Primary care is the first point of entry to the health care system and provides longitudinal relationships for patients, which is conducive to continuity of care. As such, primary care providers are well positioned to identify their patients’ need for palliative care and commence this approach early in the disease trajectory. Research shows that primary care teams are willing to provide palliative care, but experience a lack of structural supports (e.g. financial incentives, interoperable electronic medical records, etc.) to apply this approach in practice [5–8]. Moreover, practical supports, such as strategies to help with identification, coordination, and communication are also needed to help operationalize a palliative care approach into practice [9, 10]. Interactive palliative care education programs that incorporate participant discussion and/or coaching have shown promise towards effective practice change; [11–14] though most of these prior interventions were in-person, intended for a single provider profession, and/or focused on communication skills [15–18].

We developed and piloted CAPACITI (Community Access to PAlliative Care via Interprofessional Teams Intervention) as a virtual, comprehensive education program designed to provide advice, strategies, and plans of action to assist primary care teams in operationalizing an early palliative approach to care. The program was both designed and facilitated by an interprofessional team of palliative care researchers and health care professionals (physicians, nurses, social workers, etc.) to enhance the existing capacity of primary care teams without requiring ongoing financial support. CAPACITI serves to complement existing educational interventions, which teach palliative care skills, through case-based, interactive education sessions, and thus emphasizing the application of knowledge in practice. In our prior studies, CAPACITI participants reported significant increases in their identification of patients requiring palliative care, competency in providing care, and team collaboration following the intervention [19, 20]. While studies of other palliative care training interventions have demonstrated a positive influence on provider-reported outcomes, [15, 21, 22] it remains less clear how and to what extent the elements of these programs are effectively integrated into primary care team practice.

The objective of this study was to qualitatively explore the experiences of primary health care teams in Ontario, Canada who participated in the CAPACITI pilot. We sought to understand the factors that helped or hindered participating teams in applying CAPACITI components in practice. In this article, we synthesise and interpret findings from focus groups, monthly reflection survey data, and open-text survey data to determine the effectiveness of CAPACITI in supporting participants to provide an early palliative approach to care.

## Methods

### Study design and participants

We used an embedded case study approach as described by Yin [23, 24] to analyse and synthesize three sources of qualitative data collected from participating primary health care teams: monthly reflection survey data, open text survey data, and focus group transcriptions. A case study approach is highly applicable to program evaluation, allowing for the explanation, description, and exploration of multifaceted, complex processes in their natural context across multiple data sources [25–28]. The unit of analysis or phenomenon of interest in this case is the CAPACITI program and the three data sources are the embedded sub-units.

Primary health care teams working in Ontario, Canada were invited to participate in CAPACITI via primary and palliative care networks and organizations. Teams were invited to enrol in CAPACITI through advertisements across provincial primary care associations and through palliative care networks and organizations. Participating teams worked in community-based practices, had to be willing to do palliative care home visits, and were comprised of at least one prescriber (physician or nurse practitioner) and other interprofessional care providers. CAPACITI was tailored towards general practitioners, nurses, and allied health professionals as well as team administrators seeking to incorporate a palliative care approach into their practice. Before enrolling in the program, it was recommended that participants complete an educational course such as Pallium Canada’s LEAP Core, which teaches essential clinical competencies in palliative care such as symptom management, addressing psychosocial needs, and advance care planning [29]. CAPACITI was offered free to the teams, and our study did not provide any financial incentives or compensation to participants. Ethical approval for this study was obtained from the Hamilton Integrated Research Ethics Board (#7054).

### CAPACITI pilot intervention

In this initial pilot implementation of CAPACITI for primary care teams, we conducted 10 facilitated, hour-long modules once per month. All sessions were conducted virtually on-line via the Zoom Video Communications application. Each session centered around a core component of implementing a palliative care approach into primary care practice. The module topics included enhancing communication skills, early identification and assessment, team building, and engaging with caregivers and specialists (Supplemental Document 1). The development of CAPACITI was previously described in detail in a single cohort study of the pilot data [19].

**Fig. 1:**
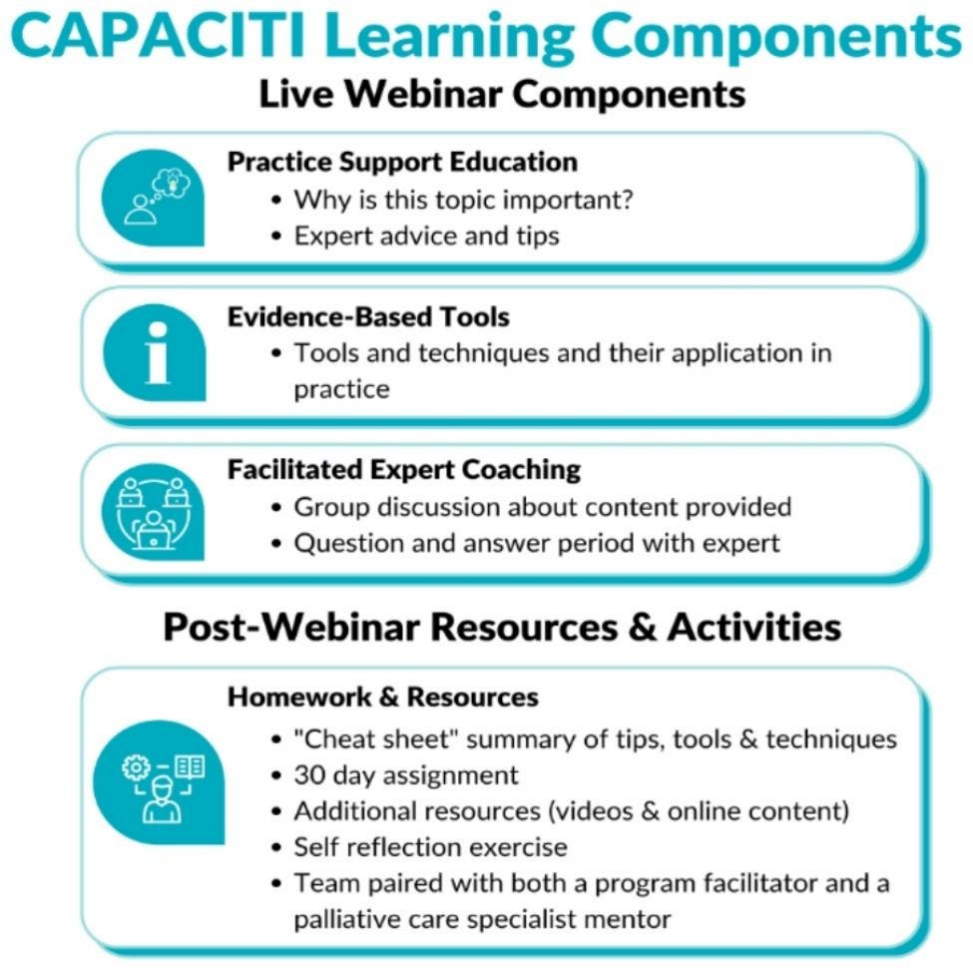
CAPACITI Learning Components

All sessions included (1) educational support for clinical practice through the provision of expert advice, (2) evidence-based tools (such as the Prognostic Indicator Guidance [PIG] which supports early identification of patients who may require a palliative approach to care), [30] and (3) coaching and facilitation to support practitioners to tailor knowledge, skills, and tools to their regional contexts. Each session began with an hour-long virtual webinar, comprised of an instructional segment followed by an interactive session with a palliative care expert clinician (palliative care physicians and nurses with 10 + years’ experience working in this specialty) who was able to answer questions, offer advice, and share their personal experiences (Fig. 1). CAPACITI included several resources to encourage the adoption of content into practice: a “cheat sheet” (a summary of the strategies presented in the webinar) and a 30-day assignment (an action to be attempted in practice, e.g., application of prognostic tools with a patient) (see Fig. 1 and Supplemental Document 1). In addition, each team was paired with a palliative care specialist mentor that they could contact for advice for the duration of the program. Teams were also assigned a CAPACITI facilitator (DB, MC, KM) who was a contact person to guide them through the program. All program resources were available for participants to download and review.

### Data collection

Data collection consisted of: 1) Written responses to a reflection survey that was completed by teams following each month-long session (two to four weeks afterwards collected via email; 2) Open text data from the midpoint (post session 6) and post intervention questionnaires asking for feedback on participating in CAPACITI, collected via an online survey (SurveyMonkey); and 3) Focus groups with individual teams at the program midpoint and post intervention (see Supplemental Document 2 for the reflection survey questions, focus group discussion guide, and open text survey data). All teams were invited to participate in a virtual focus group (Zoom teleconference) at both time points, which were conducted by trained interviewers (DB, MC, KM, HS). Focus groups were semi-structured and asked about perceptions of the program and the impact it had on the teams’ thinking and behaviour. Development of the focus group discussion guide was informed by the Consolidated Framework for Implementation Research (CFIR 2) [31]. We selected CFIR because it is one of the most used and cited frameworks to assess multilevel contextual factors in program implementation and impact [32, 33]. The five CFIR domains are intervention characteristics, outer setting (external influences), inner setting (internal factors), characteristics of individuals, and process of implementation. The focus group sessions were audio recorded, transcribed verbatim using Otter.ai software, and reviewed by the research team for accuracy. Teams that were non-responsive to data collection activities were contacted up to six times via email and/or phone call.

### Analysis

All data, including focus group transcripts, were analyzed using NVivo (version 14)qualitative analysis software. Two primary analysts (MM and VB) and a secondary analyst (DB) were involved in thematic analysis of the data. The analysts were from non-clinical backgrounds (health policy, public health, and health research methodology), with post graduate level training in qualitative research. The three analysts read through all the data and then independently coded the focus group transcripts from three teams. The data were reviewed for emergent themes and coded using the constant comparative method [34]. The analysts compared their individual findings for these three teams and consolidated coding approaches through discussion to meet consensus. Following this, a shared codebook was developed to ensure that researchers were operating on the same definitions of themes. This process was repeated by the primary analysts with the focus group transcripts for three additional teams, with the coding system revised as the analysis progressed. Once consistency in coding and thematic development was established, the primary analysts each coded half of the remaining transcripts and one analyst (MM) coded the reflection survey and open text survey data. The analysts independently reviewed and then discussed all coding, finalizing the themes derived from each data source and synthesising the overarching themes.

## Results

The CAPACITI sessions ran consecutively from January 2020 to March 2021, except for a six-month adjournment from April to August 2020 (following session 3), due to the COVID-19 pandemic. Initially, 26 primary care teams enrolled in CAPACITI. Four teams (27 participants) withdrew following the third session (March 2020) due to pandemic restructuring of their teams. Ultimately, 22 teams (159 participants) completed CAPACITI. This included: 12 Family Health Teams, 7 Community Health Centres, 2 nurse-practitioner led clinics, and 1 Aboriginal Health Access Centre. The number of participants per team ranged from 2 to 15 (median = 7). Half of the teams served rural communities, defined by Statistics Canada as communities with a core population of fewer than 10,000 people [35]. Most (75%) of the CAPACITI participants reported that they completed LEAP training prior to the program. Table 1 further describes the participant demographics.

**Table 1:** Demographic Characteristics of CAPACITI Participants (N = 159)

Profession or Role	Number	Percent
Physician	39	24.5
Registered Nurse	29	18.2
Nurse Practitioners	28	17.6
Administrator, Manager, or Case Coordinator	27	17.0
Social Worker	15	9.4
Pharmacist	7	4.4
Registered Practical Nurse	7	4.4
Dietitian	4	2.5
Other	3	1.9
Worked at Current Site		
Less than one year	30	18.9
One year to less than two years	22	13.8
Two years to less than five years	29	18.2
More than 5 years	78	49.1

Open-text survey data were collected from members of all 22 teams (2 to 13 members per team). In total, 86 team members (54.1%) provided comments at the mid-point and/or post intervention survey. Reflection data were collected from a total of 21 teams, with each team providing two to nine session specific reflections. Fifteen unique teams participated in the midpoint focus groups (8 teams) and/or final focus groups (12 teams). Each focus group involved 2 to 12 attendees (median = 5). Focus groups ranged from 25 min to an hour, with most lasting approximately 45 min.

Three core themes were identified from the three sources of data: (1) changes in practice or knowledge derived from CAPACITI, (2) utility of CAPACITI components, and (3) barriers and challenges to enacting CAPACITI in practice. These were treated as parent themes, which encompassed 12 subthemes (see Table 2). There were no notable differences in the major themes between data sources. All quotes provided are from focus groups unless otherwise specified.

**Table 2:** Summary of Themes

Themes and Subthemes	Description/Examples
**Changes in practice or knowledge derived from CAPACITI**	Ways in which CAPACITI changed (or did not change) team thinking or practice
Early identification	Changes in identifying patients who could benefit from palliative care earlier in the disease trajectory
Communication skills	Changes to communication skills within teams and with patients
Applying a palliative approach to care	General changes in applying a palliative approach to care in practice
Improved teamwork	Changes in collaborative efforts in palliative care both within teams and through outreach to external providers
**Utility of CAPACITI components**	The perceived utility of specific elements of CAPACITI.
Monthly assignments	Optional exercises for teams to become acquainted with applying CAPACITI components in practice, e.g. creation of a palliative care registry
Cheat sheet	Summary of core lessons from CAPACITI on an easy-to-reference handout
Mentorship	Consultation with an assigned palliative care expert external to team organizations to assist with learning outcomes
**Barriers and challenges to enacting CAPACITI in practice**	Internal (team- or context-based) factors caused teams to struggle with applying CAPACITI learnings in their practice
COVID-19 pandemic	Impact of the pandemic on completing CAPACITI, e.g., move to virtual-only meetings, balancing increased workload demand
Competing demands	Time constraints in completing CAPACITI components, coordinating schedules between time zones, or difficulty in scheduling mutually available times within teams
Team fragmentation	Lack of team integration, funding restrictions, and distal proximity of team members
Lack of confidence or opportunities to practice	Low individual/team comfort levels in providing palliative care, low volume of seriously ill patients to apply CAPACITI learnings
System-based challenges	Geographic limitations of access to care, system fragmentation, and a lack of team integration

### Theme 1: changes in practice or knowledge derived from CAPACITI

This theme highlights teams’ perceptions of how attending CAPACITI sessions and completing the related activities translated to changes in practice. Four subthemes emerged from the teams’ responses on advances made: (1) early identification, (2) communication skills, (3) applying a palliative approach to care, and (4) improved teamwork/collaboration.

#### Sub-Theme 1.1 early identification

Many providers expressed that CAPACITI helped them identify patients who might benefit from an early palliative approach to care. Teams reported implementing various screening tools (such as the Palliative Performance Scale or the Prognostic Indicator Guidance) or running queries in their electronic medical records (EMRs).

*“[Our] team found it helpful to have tools that can be utilized for the early identification of palliative patients. This has increased our confidence in our ability to accurately identify palliative patients from our large roster size. [We have] identified palliative patients by running an EMR query using conditions listed in the PIG* [Prognostic Indicator Guidance] *and in the supplementary material from CAPACITI (EMR algorithm). [And given our providers the] list of query results to see if they agree that the identified patients would benefit from palliative care approach.”* (Team O Reflection).

Many of the teams reviewed their caseloads with a new lens for identifying patients early in the disease trajectory rather than at end of life:

*“I felt like going through CAPACITI, [we are] definitely identifying palliative care patients earlier… Before CAPACITI, I would say, you know, pretty much end of life, I [could] count [them] on my hand… But now it’s a bit more early on identifying patients. I’ve really kind of changed.”* (Team B).

#### Sub-Theme 1.2 communication

All teams described how their approaches to communication with patients and their families changed because of CAPACITI. Participants explained the importance of initiating conversations about the disease trajectory and destigmatizing palliative care:

*“I think the communication strategies are probably the most important because [of] the understanding of what the definition of a palliative approach is if you don’t have that on your radar, or the mindset about it, then you’re going to miss a lot of these people, [and] being able to have that understanding of that approach will take the fear out of the term palliative care for patients because we’re talking about it with them as an everyday occurrence.”* (Team C).

Initiating early palliative care discussions with individuals rather than restricting these conversations to end-of-life was emphasized:

*“Identifying patients who could be potentially palliative was kind of eye-opening, so we can initiate conversations earlier rather than waiting until they’re end-of-life… and actually defining and reframing palliative care – since so many providers think palliative is end-of-life, and patients and families think palliative is end-of-life – they don’t see it as a reframing of their treatment plan. So that’s been really helpful.”* (Team W).

Some teams reflected on the importance of first gauging the patient’s readiness before initiating conversations about care:

*“The session about conversations…really struck me. [Patients] may not have a lot of information about their illness, and the prognosis and the progression. But don’t go there if they’re not emotionally able. First, you have to go with the emotional availability to want to know more. I thought that was really key in how a conversation could go sideways, and frustration from ‘well, why aren’t we talking about this.’ First you have to lay the groundwork in the emotional readiness. I thought that was great and really helpful.”* (Team I).

Teams expressed previous discomfort towards having serious illness conversations, and that better efforts were made as a result of completing CAPACITI:

*“It’s made me reflect… It might be a little bit uncomfortable, but it is worth it to have those conversations earlier so that it’s not more stressful and chaotic at the end of life when it shouldn’t be.”* (Team I).

#### Sub-Theme 1.3 applying a palliative approach to care

Many teams described how their approach to palliative care had changed, as demonstrated through their recent interactions with patients and families. Participants shared how CAPACITI had positively changed the way they think about palliative care:

*“We’re planning to make changes. We’re going to meet and talk about changing in terms of our team capacity. As an individual, it [CAPACITI] has got me thinking differently. I try to have more of those conversations about a palliative approach with people.”* (Team W).

Some participants shared that they started making appointments for their patients to meet with their primary care provider to initiate care planning conversations:

“*From the perspective of an outreach nurse… [CAPACITI] has encouraged me to try to book my clients with their primary care providers for appointments that are just going to address future planning and having those discussions separate from their regular appointments for their chronic disease management. It’s been more challenging under the context of COVID, but I’m a little more aware of doing this consciously.*” (Team U).

Further, others expressed that their approaches have become less biomedical and more informed by the patient’s own comfort levels and emotional receptiveness to having care planning discussions:

*“[We’re] getting a better sense of what patients understand about their illness and how much they would like to know [to] allow a more collaborative and patient centred approach. [Our] providers are more willing to wait and to not try and fill in the blanks but make more space for clients to describe what is important to them at that moment.”* (Team I Reflection).

*“Whenever I see a patient with a life limiting illness, even if it is very early on, I think through the tools and I think like the within a year tool, or the surprise question [i.e., would you be surprised if this patient were to die in the next year?* [36]], I think of those now, every single time, which I hadn’t been doing before. So even though it’s not always formal, I don’t document on it, or I don’t put them on the registry that we did create, I think about that a lot more, which has been very helpful.” (Team E).

#### Sub-Theme 1.4 improved teamwork

Most teams reported greater collaborative efforts within their own primary care teams and through outreach to relevant specialists and community-based organizations. Previously, some teams expressed that while team members were independently practicing a palliative care approach, a coordinated strategy was absent. CAPACITI inspired the adoption of a more unified approach:

*“In the past our providers didn’t have a clear understanding that they could connect with our local specialists for palliative care consultation. Some of our providers are of the mind set to let the specialist do their job and the family physician will do theirs. CAPACITI helped them [our team members] understand that it’s a team effort and have engaged with clients more to increase communication with specialists.”* (Team T Reflection).

Efforts to strengthen interdisciplinary care reduced system fragmentation and repetition of information across multiple sources:

*“It is essential for us to build a multidisciplinary team that has a clear communication protocol when it comes to patient care. A team that communicates consistently to [the] patient and establishes regular goals eliminates the potential of repetition in obtaining information.”* (Team U).

*“Our team is becoming more excited, cohesive, and understanding of the vision of the palliative care team we are foreseeing in the future. Each session brings one more piece of the missing puzzle, and a concrete vision and plan are forming.”* (Team Y).

### Theme 2: utility of CAPACITI components

This theme describes the perceived usefulness of CAPACITI components in primary care practice. The format and content were generally well regarded by teams. Three main components of CAPACITI were consistently outlined by respondents: the 30-day assignments, sessional cheat sheets, and arranged mentorship with a palliative care specialist.

#### Sub-Theme 2.1 monthly assignments

The most widely implemented 30-day assignment was from the second session. This assignment asked participants to create a registry to identify patients in need of a palliative approach to care. Eight teams shared that they had been successful in establishing a palliative care registry within their respective practices.

“*The registry was good to build so that we know which patients are maybe pre-palliative or tolerated palliation early [in their illness trajectory].*” (Team B).

Apart from establishing a registry, other assignments reported as being attempted were application of the communication tools (Session 5) and scheduling team meetings to discuss components of CAPACITI and create an operationalization plan (Starting session 1).

#### Sub-Theme 2.2 cheat sheets

The cheat sheets were highly regarded by the teams. Many viewed these primers as a helpful summary of CAPACITI’s lessons and a way to share this information with team members who were unable to attend the session webinar:

“*The cheat sheets… were a great summary of everything that was discussed. It was a great way to communicate to physicians who were not able to attend the meeting.*” (Team N).

#### Sub-Theme 2.3 mentorship

The nature and perceived utility of the relationship with the assigned palliative care specialist mentor varied across teams. Most teams did not connect with their mentor as much as they had hoped, and some did not use their mentor at all. This was, in part, due to scheduling conflicts or shifts in practice because of the pandemic. Some teams explained that they did not have any patient encounters where they felt it necessary to engage the mentor. We also offered access to a forum of palliative care experts where the teams could pose their CAPACITI related questions, however no teams used the platform.

“*I think we talked to our mentors once. We probably could have reached out to her. But we never really had any big questions that we needed to reach out for.*” (Team V).

### Theme 3: Barriers and challenges to enacting CAPACITI in practice

Teams reported barriers to participating in CAPACITI that also posed as challenges to operationalizing the program material in practice. Challenges that were often discussed included the COVID-19 pandemic, competing demands, funding limitations and team fragmentation, lack of confidence or opportunities to practice, and team or system-based issues.

#### Sub-Theme 3.1 COVID-19 pandemic

CAPACITI was paused for five months at the beginning of the pandemic. As such, the COVID-19 pandemic was cited by almost all teams as a strong impediment against attending CAPACITI sessions, completing assignments, and adopting content into practice. Teams highlighted the pandemic’s impact on their ability to meet in person as a team, discuss, and participate in CAPACITI:

“*We ran into some struggles because of not being able to be together all the time and doing certain things because of this pandemic. So, for some of the challenges [activities], we were able to do them as best as we could, but maybe not to the fullest.*” (Team N).

*“All efforts around CAPACITI have become very difficult since onset of COVID in March as MDs and staff have been redeployed to various degrees.”* (Team A Open Text Survey).

#### Sub-Theme 3.2 Competing demands

Several teams shared that competing demands and having a lack of time were significant obstacles in completing CAPACITI. A few teams indicated that motivation to finish the program wavered towards the end, largely due to CAPACITI continuing for over a year and the teams experiencing internal changes during this expanse of time. The pandemic exacerbated time restraints in the unprecedented shift to remote work due to social distancing protocols and the need for teams to redeploy their staff to manage different priority areas. For many teams, CAPACITI became a low priority. Teams also cited difficulties in finding mutually available times for them to go through CAPACITI materials, as well as general competing interests in primary care, regardless of the pandemic:

“*I think that the challenge… was just being able to implement the [lessons] and having the time to sit down and discuss how we’re going to implement things. There were a lot of competing interests. There were lots of challenges aside from this particular project for the organization… it would have been nice to have been able to devote a lot of our time to CAPACITI.*” (Team G).

#### Sub-Theme 3.3 Team fragmentation

Some teams described that funding limitations and lack of team integration, role clarity, and interprofessional communication were barriers to their participation and adoption of content into practice, especially in rural areas. The physical distance between the members of some teams presented a barrier to coordinating and participating in CAPACITI activities:

“*A barrier was role clarification and continuing to understand the purpose of CAPACITI and how the program will help us develop structure and function as a team within our large organization, especially since most providers work across different offices.*” (Team O).

“*The main barrier our team encountered was the communication issue… we were not able to communicate effectively with other teams because of geographical location and time constraints. This was a major obstacle.*” (Team U).

Teams also discussed internal issues, such as how competing interests between team members and/or lack of team collaboration, posed a challenge to fully participating in CAPACITI:

“*We as a team needed to commit to doing that [CAPACITI], because it is very easy to just put it off to the side. So, we really need to strategize a way to make sure that it is and stays relevant and in front of us the whole time.*” (Team I).

#### Sub-Theme 3.4 Lack of confidence or opportunities to practice

Participants expressed discomfort in placing the palliative ‘label’ on patients, particularly due to the implication of end-of-life or believing that it may be too early in a patient’s disease trajectory to introduce this approach:

“*I do think that there is always a hesitation to put that person into that box… There is a huge hesitation, and I’m thinking maybe it’s too early to do that. I don’t know. It wouldn’t surprise me at all if the rest of the team wouldn’t even be thinking of [a patient] as palliative.*” (Team Z).

Some teams shared that they did not see many patients in their daily practices that could benefit from a palliative approach to care, and therefore did not have the opportunity to practice their skills:

“*The biggest barrier is clinical confidence when dealing with more complicated [palliative care] cases. [It’s] one thing if you do it every day, but at the frequency I’m doing it, it’s always like I have to look it up all over again.*” (Team G).

#### Sub-Theme 3.5 System-based challenges

Several teams described barriers including lack of system integration and distance from other care settings and providers, particularly in rural locations. Certain teams expressed obstacles inherent to their location such as the nearest pharmacy being over 2-hours away or that the closest specialists and doctors were over 500 kilometres away or outside of the province, thereby hindering opportunities for interprofessional collaboration.

“*We take for granted that we have all these services available, and we can call on them… but getting everyone to work toward the same goal is a challenge for us, and we continue to try to address it.*” (Team G).

“*There’s a few of those system barriers as well…some of those silos still exist. It makes it a bit of a challenge to accomplish some of those goals set out.*” (Team X).

## Discussion

We examined three sources of qualitative data to gain an in-depth understanding of what elements of the CAPACITI education intervention participating teams found useful and how they incorporated this acquired knowledge into practice. Three major themes were generated from our analysis: changes in practice or knowledge derived from CAPACITI, utility of CAPACITI components, and barriers and challenges to enacting CAPACITI in practice. Participants shared that CAPACITI helped them change their processes and behaviours, which included earlier identification of those who may benefit from a palliative approach to care and initiating serious illness conversations. Operationalization of other course content was less evident, possibly because teams worked on the early steps of CAPACITI but did not have a chance to focus on subsequent topics, or that efforts made towards these respective actions, for example proactive care planning, are difficult to articulate.

By instituting key elements of successful health care professional training programs demonstrated in existing literature, [37–39] the goal of CAPACITI was to translate knowledge into care via interactive sessions, ultimately, empowering teams to apply these principles within their context-specific practices. The tools, cheat sheets, and virtual format, along with the topics covered, were regarded by teams as effective elements of the program. CAPACITI encouraged processes to enhance ongoing interprofessional collaboration within the teams, including group problem solving and planning, information sharing, communication strategies, mapping of internal and external partners, and role determination. The utility of CAPACITI is supported by findings from systematic reviews that behaviours are most successfully changed by educational interventions that are participatory, use blended teaching modalities, synthesize learner reflection, and provide support for decision-making [18, 40–43]. Teams attested that a strength of the program was the aim to enhance interprofessional collaboration and encourage team-based planning, beginning with the first session, “Building a Strong Team”. This objective aligns with evidence that training which facilitates interprofessional team-based care towards a learner-focused quality improvement plan, has the greatest potential for changing practice [22, 40, 44].

CAPACITI’s emphasis on strengthening interprofessional collaboration within primary care teams seemed to enhance continuity of care by helping teams to establish various means of communication and information sharing, as well as role clarity [45, 46]. Prior studies have demonstrated that feelings of inclusion and an understanding of member’s respective roles are important enablers in promoting effective interprofessional practice within primary care teams [47–49]. Through CAPACITI, teams also gained comfort reaching out to external organizations and specialists in the community to solicit advice or to facilitate continuity of care between providers. Our findings are substantiated by research which demonstrates the contribution of interprofessional team collaboration towards providing a comprehensive approach to managing complex conditions, thus enhancing the quality of patient care and subsequent health outcomes [50, 51].

Finally, to reach a wide audience of health care teams across Ontario in an accessible manner, CAPACITI was exclusively facilitated online. Several studies have reported that e-learning is one of the most successful tools to facilitate knowledge acquisition among health care providers due to its flexibility, accessibility, and ability to meet evolving and diverse educational needs [42]. The importance of a virtual format that promotes interactive learning and motivates participants to engage with others regarding the knowledge translated, has also been noted, particularly in teaching collaborative practice [52, 53].

### Barriers

Barriers to knowledge translation have also been well described in the literature [41, 42, 54]. Some of these challenges were experienced and highlighted by CAPACITI participants, including lack of time, resources, team coordination, and system level cohesion. The program was intended to build on existing team capacity towards instilling a palliative approach to care, without requiring additional funding or resources. We posited that by changing the way health care providers think about palliative care and helping them to apply some tested strategies, a palliative approach to care can be integrated into practice. Many of the teams in CAPACITI demonstrated proof of this concept through identifying patients that could benefit from palliative care earlier in the disease trajectory, improved communication skills and comfort levels discussing palliative approaches to care with patients and enhancing teamwork. Nonetheless, training and implementation diverts time from regular work activities, which became more challenging with the advent of the pandemic. Strong buy-in and motivation is vital to changing practice, [55] qualities which were inconsistent among members and leadership in some teams. The barriers mentioned could also adversely impact job satisfaction and retention, impeding team-based knowledge transition activities and empowerment to implement change [56, 57]. Some teams reported that they did not have the patient volume to become proficient with the skills taught. Others mentioned low confidence or worry about the emotional reaction when introducing the idea of “palliative care” with patients [58–60]. Despite these barriers, including those exacerbated by the COVID-19 pandemic, all teams that completed CAPACITI expressed that their involvement in the program resulted in positive changes in their thinking and approach towards palliative care.

### Considerations

Our pilot study highlights aspects in the delivery of evidence-based content that require further examination, namely optimal program duration and the role of facilitation to enhance learning and behavior change. Although longitudinal palliative care education interventions that run for a year or longer are not uncommon, [15, 61] the 10-month duration of CAPACITI, compounded by the six month break at the start of the COVID-19 pandemic, seemed too long to keep all teams fully engaged. We implemented the modules leaving one-month in between to allow teams time to apply the teachings in practice. However, some found this time lengthy, making it difficult to maintain momentum, while others needed more time to complete the activities [62]. To shorten the length of CAPACITI while preserving the content and maintaining a stepwise implementation plan, we would divide the 10 session pilot program into three shorter modules that would each take 3 months to complete and could be taken solely or in succession. Secondly, we offered facilitated sessions in an attempt to tailor the content of CAPACITI to the needs and context of the teams, using a virtual platform. There was an acceleration in the advent of self-directed online education with highly contextualized content as a result of the pandemic [63–65 A purely self-directed format may prove to be a cost-effective alternative to the live facilitated sessions we offered in CAPACITI and potentially preferred by participants as being more convenient. The findings from this pilot study will be applied to the development of a national randomized controlled trial to compare the effectiveness of self-directed education alone versus education with facilitation, in the delivery of a three-part, revised version of CAPACITI [62].

### Limitations

A key limitation of this study is that not all teams participated in a focus group interview. Although we collected qualitative data from all teams, it is likely that those willing to participate in a focus group were more engaged in the CAPACITI program than those that did not, which may have biased our findings. Another limitation to the study was the effect of the COVID-19 pandemic on CAPACITI, which caused the program to be put on temporary hiatus due to the teams shifting their priorities. COVID-19 redeployment, team members working together less due to social distancing safety measures, and the extended length of CAPACITI likely contributed to participant attrition, hindered uptake of the program content into practice, and limited team collaboration. Despite this, most of the teams remained committed to CAPACITI and shared positive takeaways of their experiences. However, there may be bias in responses from those hesitant to mention issues that would reflect unfavourably on their fellow team members in a focus group setting. Rigorous testing of CAPACITI as a randomized controlled trial is warranted to determine the perceived benefits of facilitated education over a self-directed approach.

## Conclusion

CAPACITI is a multicomponent educational program, designed to build palliative care capacity within primary care teams, intended be applied synergistically alongside other clinical training initiatives. Our qualitative analysis of this pilot program demonstrated that CAPACITI, and specifically this facilitated approach to knowledge translation, helped primary care teams develop the tenets towards applying a palliative approach to care. As we implement this program in diverse contexts, it is important to be aware of how intra-team interactions influence power dynamics and role clarity when providing care in a collaborative context. Future iterations of CAPACITI and other training initiatives also need to help mitigate local barriers, such as team fragmentation and system-based challenges to encourage interprofessional collaboration and knowledge translation. Team climate and readiness for change are also important aspects of team functioning and knowledge uptake to be considered.

### Electronic supplementary material

Below is the link to the electronic supplementary material.

Supplementary Material 1

## Data Availability

The datasets used and/or analyzed during the current study are available from the corresponding author on reasonable request.
