# Supplementary material for: Implementing palliative care education into primary care practice: a qualitative case study of the CAPACITI pilot program
Source: BMC Palliat Care. 2023 Sep 28;22:143. doi: 10.1186/s12904-023-01265-7 (PMC10537555; doi:10.1186/s12904-023-01265-7)
Supplement: Supplementary file 1 — Supplementary Material 1 [file 12904_2023_1265_MOESM1_ESM.docx]

**Supplemental Document 1: Topics and Content of the CAPACITI Program**

| **Session Topics** | **Session Objectives** | **Tools and Resources*** | **30-Day Assignments** |
| --- | --- | --- | --- |
| **S1**  How to get Started: Building a Strong Team | ● Describe evidence related to building strong teams  ● Assess your team’s readiness to get started | ● Sentinel Event Analysis Tool | ● Discuss a Sentinel Event and reflect on patients who’d benefit from an early palliative care approach. |
| **S2**  Identification and Monitoring of Patients at End of Life | ● Describe tools to identify those who’d benefit from a palliative care approach – start with those at end of life  ● Describe strategies to track and monitor identified patients | ● Supportive and Palliative Care Indicators Tool (SPICT)  ● Prognostic Indicator Guidance (PIG) tool (Ontario adaption)  ● Palliative Care registry examples | ● Identify patients that could benefit from a palliative care approach.  ● Develop a plan for on-going identification  ● Create a registry |
| **S3**  Identification and Monitoring of Patients at Early /Transitional Stages | ● Describe tools /processes to identify (and track) patients who are in a transitional stage and could benefit from a palliative care approach | ● Same as S2 | ● Identify early and transitional patients in your practice requiring a palliative care approach and adjust strategy for monitoring them in a registry or list |
| **S4**  Communicating with Patients and Families about Serious Illness: Part 1 | ● Describe the value of and how to initiate open, early conversations with patients and families about serious illness | ● Model of person-centered conversation  ● Goals of Care model video  ● Speak up Ontario guide to advance care planning  ● Serious Illness Conversation Guide (Ariadne Labs) | ● Watch the Goals of Care model conversation video |
| **S5**  Communicating with Patients and Families about Serious Illness: Part 2 | ● Describe a process to incorporate having conversations about serious illness into practice | ● Same as S4 | ● Have an “open” conversation with a few patients with a serious illness and create a strategy in your practice for having these conversations |
| **S6**  Assessing Patient Needs | ● Describe clinical practices that support assessment of transitional and EOL patients  ● Describe a process for on-going assessment | ● Palliative Performance Scale (PPS) tool  ● Clinical Frailty Scale  ● Distress Thermometer  ● Edmonton Symptom Assessment System (ESAS)  ● Canadian Problem Checklist | ● Assess the holistic needs of a few patients you identified |
| **S7**  Care Planning with the Patient | ● Describe everyday clinical practices that support proactive care planning for transitional and EOL patients | ● Research paper describing triggers for upstream identification, proactive practices, and broadened care team | ● Create a plan to shift to proactive care planning strategies |
| **S8**  Care Planning with the Broader Care Team | ● Describe ways to use the broader network of local resources and customize strategies for after-hours care with broader team | ● Examples of contact lists of external partners | ● Create a community resource list with contact information  ● Create an afterhours /urgent care plan |
| **S9**  Engaging the Family Caregiver | ● Recognize the role of the family caregiver  ● Describe ways to activate the patient’s informal care team (e.g. caregiver) | ● Carer Support Needs Assessment Tool (CSNAT) UK  ● Virtualhospice.ca discussion forums for caregivers | ● Assess the needs of a few caregivers and explore how the caregiver can be part of the care team |
| **S10**  Engaging with Specialists and Sustaining | ● Determine how/when to interact with: i) disease specialists; ii) local palliative care specialist | ● Who, When, and How to ask for help primer to engaging specialists | ● Consult with disease / palliative care specialist |

* A summary sheet is provided for each session as a quick reference guide to assist with the monthly assignment and implement the process in practice, in general.

**Supplemental Document 2: CAPACITI Reflection Survey, Focus Group Guide, and Open Text Questions**

**A. CAPACITI Reflection Survey Items**

1. What was the most helpful part of this session?
2. How could this session be improved?
3. Provide concrete examples of ways your team changed or will change its approach, practice, or work due to completing this session.
4. Describe any barriers your team encountered in implementing these changes into practice and how these were overcome:
5. How has this session helped you to improve care for patients/caregivers?

**B. Focus Group Questions:  *Thinking about all the CAPACITI sessions (1 to 10)***

**Part 1: Suitability/Utility of the CAPACITI program**

A. What did you like or not like about the CAPACITI program?

Prompts for discussion are: i) monthly activities (challenges) assigned to teams,

ii) support or active facilitation from the research team, and

iii) mentor involvement (ever called/used?)

**Part 2: Part 2: Utility of the CAPACITI program**

***As a result of completing CAPACITI what do you now do differently, either as an individual or as a team?***

*Think about ways in which you changed your…*

- *Thinking*
- *Behaviour*
- *Processes*

***Which tips given in the CAPACITI sessions were the most impactful? The least?***

**Part 3: Operationalization of CAPACITI challenges**

***Thinking about the monthly activities (challenges), which of these did you find useful and why or why not?***

*Which were you able to complete? What did you do (details)?*

*What things posed as barriers or facilitators?*

1. *Impact of structural factors? [funding model, region, population (rural/urban), incentives]*
2. *Impact of process factors? (Team culture, attitudes, common vision, composition)*

*Think beyond COVID!*

**Closing questions**

Thinking about the whole CAPACITI program, what would you keep or change? What are your recommendations?

Is there anything else you would like to tell us about you and your “team’s” experience with the program?

**C: Open Text Survey Questions**

1. Approximately how long have you worked at your current site or practice?
2. Thinking about the last CAPACITI session, please provide examples of things you or your team did as a result of this session.
3. Describe any barriers your team encountered in the past session.
4. Please type any additional comments or points of clarification you wish to add in regard to your [qualitative] responses.
